# Health Abroad: A Medical Handbook of Travel

**Published:** 1900-12

**Authors:** 


**Health Abroad: A Medical Handbook of Travel.** Edited by Edmund Hobhouse, M.D. Pp. xv., 372. London: Smith, Elder, & Co. 1899.

The main object of this book is to enable the traveller to preserve his health whilst travelling in unknown climates, to be familiar with the various emergencies which may arise when medical aid is not forthcoming, and to treat them to the best advantage. Well does the editor preface the contents of the volume with the remark that "Too often foreign travel, instead of restoring health, is the means of sowing the seeds of disease in those previously strong and healthy, when a couple of days' rest or a few simple precautions might have saved a life from being wrecked by chronic ill-health or destroyed by fever." The wide scope of the subject matter has rightly led the responsibility for the information on the various districts being placed in the hands of those who can speak from personal experience gained by residence: thus for Northern Africa and Egypt, Dr. Leigh Canney is the author; for Central Africa, Dr. Harford Battersby; and so forth. The article on South Africa is not quite up to date for obvious reasons, but we hope that the sanitation of Pretoria, the capital of the "Transvaal," will not long "leave much to be desired."

The book is eminently handy, and the contents well arranged, and of great practical utility to all who contemplate leaving their native land.

